# Effect of early sleep apnoea treatment with adaptive servo-ventilation in acute stroke patients on cerebral lesion evolution and neurological outcomes: study protocol for a multicentre, randomized controlled, rater-blinded, clinical trial (eSATIS: early Sleep Apnoea Treatment in Stroke)

**DOI:** 10.1186/s13063-020-04977-w

**Published:** 2021-01-22

**Authors:** Simone B. Duss, Anne-Kathrin Brill, Sébastien Baillieul, Thomas Horvath, Frédéric Zubler, Dominique Flügel, Georg Kägi, Gabriel Benz, Corrado Bernasconi, Sebastian R. Ott, Lyudmila Korostovtseva, Yurii Sviryaev, Farid Salih, Matthias Endres, Renaud Tamisier, Haralampos Gouveris, Yaroslav Winter, Niklaus Denier, Roland Wiest, Marcel Arnold, Markus H. Schmidt, Jean-Louis Pépin, Claudio L. A. Bassetti

**Affiliations:** 1 Department of Neurology, Bern University Hospital (Inselspital) and University of Bern, Bern, Switzerland; 2 Interdisciplinary Sleep-Wake-Epilepsy-Center, Bern University Hospital (Inselspital) and University of Bern, Bern, Switzerland; 3 Department of Pulmonary Medicine, Bern University Hospital (Inselspital) and University of Bern, Bern, Switzerland; 4 Grenoble Alpes University, HP2 Laboratory, INSERM U1042, Grenoble, France; 5 Pôle Thorax et Vaisseaux, Grenoble Alpes University Hospital, Grenoble, France; 6 Department of Neurology, Cantonal Hospital St. Gallen, St. Gallen, Switzerland; 7 Department of Pneumology, Cantonal Hospital St Gallen, St. Gallen, Switzerland; 8 Hypertension Department, Somnology Group, Almazov National Medical Research Centre, St. Petersburg, Russia; 9 Department of Neurology, Center for Stroke Research Berlin (CSB), Charité – University Medicine Berlin, Berlin, Germany; 10 Department of Otorhinolaryngology, Medical Centre of the Johannes Gutenberg University, Mainz, Germany; 11 Department of Neurology, Medical Centre of the Johannes Gutenberg University, Mainz, Germany; 12 University Institute of Diagnostic and Interventional Neuroradiology, Bern University Hospital (Inselspital) and University of Bern, Bern, Switzerland; 13 Department of Neurology, Sechenov University, Moscow, Russia

**Keywords:** Sleep-disordered breathing, Stroke, MRI evolution, Treatment, Adaptive servo-ventilation, Randomized controlled trial, Outcome

## Abstract

**Background:**

Sleep-disordered breathing (SDB) is highly prevalent in acute ischaemic stroke and is associated with worse functional outcome and increased risk of recurrence. Recent meta-analyses suggest the possibility of beneficial effects of nocturnal ventilatory treatments (continuous positive airway pressure (CPAP) or adaptive servo-ventilation (ASV)) in stroke patients with SDB. The evidence for a favourable effect of early SDB treatment in acute stroke patients remains, however, uncertain.

**Methods:**

eSATIS is an open-label, multicentre (6 centres in 4 countries), interventional, randomized controlled trial in patients with acute ischaemic stroke and significant SDB. Primary outcome of the study is the impact of immediate SDB treatment with non-invasive ASV on infarct progression measured with magnetic resonance imaging in the first 3 months after stroke. Secondary outcomes are the effects of immediate SDB treatment vs non-treatment on clinical outcome (independence in daily functioning, new cardio-/cerebrovascular events including death, cognition) and physiological parameters (blood pressure, endothelial functioning/arterial stiffness). After respiratory polygraphy in the first night after stroke, patients are classified as having significant SDB (apnoea-hypopnoea index (AHI) > 20/h) or no SDB (AHI < 5/h). Patients with significant SDB are randomized to treatment (ASV+ group) or no treatment (ASV− group) from the second night after stroke. In all patients, clinical, physiological and magnetic resonance imaging studies are performed between day 1 (visit 1) and days 4–7 (visit 4) and repeated at day 90 ± 7 (visit 6) after stroke.

**Discussion:**

The trial will give information on the feasibility and efficacy of ASV treatment in patients with acute stroke and SDB and allows assessing the impact of SDB on stroke outcome. Diagnosing and treating SDB during the acute phase of stroke is not yet current medical practice. Evidence in favour of ASV treatment from a randomized multicentre trial may lead to a change in stroke care and to improved outcomes.

**Trial registration:**

ClinicalTrials.gov NCT02554487, retrospectively registered on 16 September 2015 (actual study start date, 13 August 2015), and www.kofam.ch (SNCTP000001521).

**Supplementary Information:**

The online version contains supplementary material available at 10.1186/s13063-020-04977-w.

## Background

Sleep-disordered breathing (SDB) is a highly prevalent comorbidity in acute ischaemic stroke patients [1, 2]. About two-thirds of this patient group suffers from any degree of SDB, up to 40% present an apnoea-hypopnea index (AHI) above 20/h and one third even suffers from a severe form with an AHI > 30/h [1–4].

A bidirectional relationship between SDB and stroke can be assumed [5, 6]. SDB, especially the obstructive subtype, is not only an independent risk factor for stroke (e.g., [7]) but is also associated with a more rapid progression of stroke severity, with higher blood pressure levels and longer hospitalization in the acute phase [8–10]. Chronically, stroke patients with comorbid SDB exhibit worse functional outcome and higher mortality [11, 12]. Several pathophysiological conditions associated with SDB are believed to cause harmful effects [13]. These include intermittent hypoxemia, intrathoracic pressure changes and sympathetic activation, which may trigger cardiac arrhythmias or blood pressure swings [14, 15], potentially leading to harmful hypo- or hyper-perfusion of acutely damaged brain tissue. The ischaemic penumbra may be amenable to reperfusion. Consequently, any treatment stabilizing cerebral perfusion and preserving constant brain tissue oxygenation may be helpful [16].

Effective methods to normalize obstructive SDB are available, including continuous positive airway pressure (CPAP). However, central SDB is also often present in acute stroke. Some studies even suggest that new-onset SDB is often of central type [17, 18]. In particular, central apnoeas and central periodic breathing were also reported in patients with unilateral lesions of variable topography without a disturbed level of consciousness or overt heart failure, which potentially involve autonomic or volitional brain areas participating in respiratory control [19]. Adaptive servo-ventilation (ASV) is considered superior to CPAP in correcting both central and obstructive SDB [20]. Meta-analyses on the initiation of SDB treatment in the very first days following stroke postulate a positive association with neurofunctional improvement with CPAP but with a considerable heterogeneity across the included trials [21]. Prior studies reporting a positive impact of CPAP treatment after acute stroke mainly included TIA patients, milder strokes and younger patients. More recently, Bravata and colleagues found better neurological functioning at about 1 year post stroke, assessed with the modified Rankin scale (mRS) and the National Institute of Health Stroke Scale (NIHSS), in ischaemic stroke patients with obstructive sleep apnoea (OSA) and compliant CPAP use but not in the intention-to-treat analysis [22]. In this study, CPAP was initiated with a median time delay of about 2 months from onset of stroke symptoms. These studies have not assessed the acute neuroradiological evolution or change in network connectivity in the acute stroke phase under ASV treatment versus no treatment.

Thus, eSATIS aims to investigate the effects of immediate treatment of significant SDB (AHI > 20/h) with non-invasive ASV started within 48 to 68 h after stroke onset on the ischaemic lesion evolution assessed by magnetic resonance imaging (MRI) 3 months after the event. The secondary trial objectives assess whether immediate onset of ASV treatment in stroke patients with SDB improves clinical stroke outcomes and physiological parameters. The overall impact of significant SDB compared to no SDB on theses outcomes is also assessed.

## Methods

### Study design and setting

The present study is a prospective, multicentre, randomized, open-label, rater-blinded clinical trial in ischaemic stroke patients with SDB (AHI > 20/h, approximate recruitment goal *n* = 134), including a control cohort of stroke patients without SDB observed for the same duration of time (no SDB; AHI < 5/h, approximate recruitment goal *n* = 66). The study is conducted at 6 study sites in 4 countries: Bern University Hospital (Switzerland), Cantonal Hospital St. Gallen (Switzerland), Almazov National Medical Research Centre St. Petersburg (Russia), Grenoble Alpes University Hospital (France), Medical Centre of the Johannes Gutenberg University Mainz (Germany) and Charité - Universitätsmedizin Berlin (Germany). It was designed and initiated by an interdisciplinary research team of neurologists, neuroradiologists and pulmonary physicians at the Bern University Hospital and is coordinated by this centre.

The trial was registered on ClinicalTrials.gov (NCT02554487) on 16 September 2015 1 month after recruitment of the first patient (first patient in 13 August 2015) as well as on www.kofam.ch (SNCTP000001521). The initially monocentric trial became multicentre in May 2017 as the second centre in Switzerland, St. Gallen, was initiated and was then successively extended to Mainz, Berlin, St. Petersburg and Grenoble. Feasibility of the trial at each centre was evaluated based on the number of admitted stroke patients to a centre, availability of required infrastructure and qualified staff in the necessary disciplines to conduct the trial: neurology, pulmonary medicine and/or sleep medicine as well as neuroradiology. Feasibility of diagnosing and treating SDB in acute stroke patients was shown in former clinical studies of the Bernese study team (SAS CARE 1 & 2, [23–25]).

Screening and recruitment procedures for the current trial (described in the following paragraph) were established and evaluated within clinical routine at the coordinating centre in Bern.

### Screening and recruitment of participants

We recruit patients suffering from an ischaemic stroke in the acute phase of stroke (hospitalization within 24 h of symptom onset). Potential participants are identified at admission to the emergency and stroke units of the 6 participating study sites following review of patients’ electronic medical records. Screening and recruitment requires the presence of a study physician, the non-invasive ventilation and MRI team and is usually performed from Sunday night until Friday morning. Inclusion and exclusion criteria are outlined in Table 1 and are checked according to a two-step screening algorithm provided to all centres by the coordinating centre of Bern.

**Table 1:** Participant inclusion and exclusion criteria and criteria for randomization

*Inclusion criteria*	*Exclusion criteria*
1. Informed consent as documented by signature 2. Admission to one of the participating centres 3. Age 18–85 years 4. Ischaemic stroke detectable by neuroimaging, affecting internal carotid artery, anterior cerebral artery (ACA), middle cerebral artery (MCA), posterior cerebral artery (PCA) and/or branches thereof 5. Symptom onset to admission < 24 h 6. AHI > 20/h or < 5/h	1. Primary haemorrhagic stroke or secondary parenchymal haemorrhage (PH 1 and PH 2 according to ECASS) 2. Small strokes (diameter < 1.5 cm) 3. Coma/Stupor 4. Clinically unstable or life-threatening condition 5. Heart failure defined as congestive heart failure (CHF) functional class NYHA III-IV **OR** CHF NYHA II **and** hospitalization caused by CHF in the preceding 24 months **OR** left ventricular ejection fraction ≤ 45% 6. Intubation or oxygen supply > 2 L/min. 7. Known progressive neurological diseases 8. Drug or alcohol abuse 9. Inability to follow study procedure 10. Pregnancy 11. Any given contraindications to MRI or MRI-contrast agent 12. Any given contraindications to ASV treatment 13. Protocol addendum made on 28.05.2020: Exclusion of patient with clinical symptoms of COVID-19 infection (fever, signs of acute respiratory infection, loss of taste or smell) during hospitalization due to acute stroke*
*Randomization criteria*	
1. AHI > 20/h	

*AHI* apnoea-hypopnoea index, *ASV* adaptive servo-ventilation, *ECASS* European Cooperative Acute Stroke Study, *MRI* magnetic resonance imaging, *NYHA* New York Heart Association

*Notes: COVID-19 testing algorithms of each centre apply and each centre must adhere to their country’s official standards and government guidelines

#### First step

The study physician selects patients eligible for respiratory polygraphy depending on patients’ age (18–85 years), stroke location (supratentorial), stroke size on diagnostic imaging and state of consciousness and respiratory independence (no coma/intubation). After this first screening step and depending on centres’ standard operating procedures, a respiratory polygraphy is prescribed within clinical routine or as part of the trial in the first night after stroke. Accordingly, a study physician collects the patient’s or his/her next-of-kin’s/legal representative’s informed consent before or after the respiratory assessment.

#### Second step

**Fig. 1:**
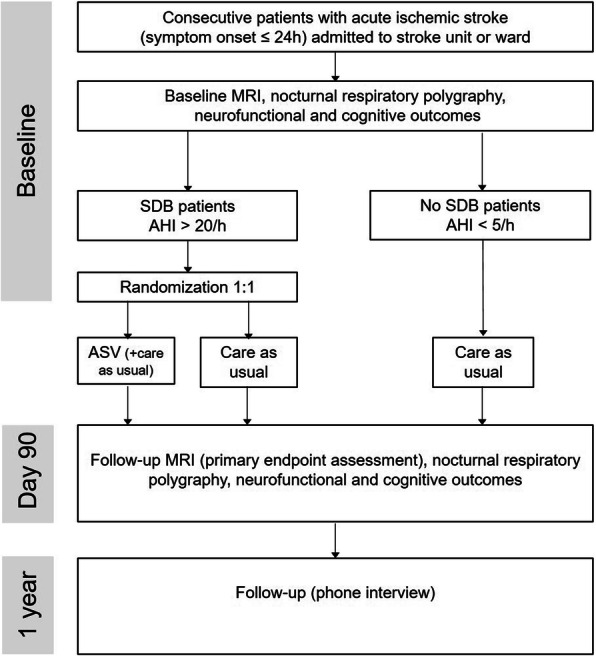
Study flow chart. Ischaemic stroke patients admitted within 24 h of symptom onset undergo respiratory polygraphy on night 1. Patients with AHI > 20/h are randomized to ASV versus no ASV treatment. Patients with AHI < 5/h are followed in parallel. New cerebro-cardiovascular events or death are assessed after 1 year by telephone interview. Abbreviations: AHI, apnoea-hypopnoea index; SDB, sleep-disordered breathing; ASV, adaptive servo-ventilation

Based on the result of the respiratory polygraphy, the lesion diameter confirmed on the MRI at day 1, and after the study physician has checked the remaining exclusion criteria listed in Table 1, eligible patients with significant SDB (AHI > 20/h) are randomized to ASV treatment (SDB ASV+) versus no ventilation treatment (SDB ASV−) applied between the 2nd and 89th night following stroke. Eligible patients with an AHI < 5/h can be included into the control cohort without SDB (noSDB group). Figure 1 gives an overview on the study design.

Every centre uses the same screening log. To identify the main reasons why patients are not eligible and to take action in case of potential screening and recruitment difficulties, each centre sends its anonymized screening log to the coordinating study team in Bern on a regular basis. Based on the screening experience in Bern before the trial became multicentre, eligibility criteria were adapted to increase the number of potentially eligible patients and generalizability of results. The main adaptations included a reduction of inclusion AHI from 30/h to 20/h, extension of upper age limit from 80 to 85 and elimination of NIHSS > 4 as an inclusion criterion. These modifications are trackable on clinicaltrials.gov.

Beside bi-annual investigator meetings, trial progression is also evaluated by the Swiss National Science Foundation (SNSF), the main financial supporter of the trial, based on bi-annual progress reports provided by the coordination centre of Bern.

To ensure retention and follow-up of each patient, follow-up visits are planned together with the patients and where possible combined with clinical routine visits. A study nurse reminds patients of their onsite visit by a phone call or letter and organizes the transport from their home or rehabilitation unit to the hospital if necessary. We also provide patients with our contact information (e-mail, phone) and encourage them to call in case of any also medical question.

### Sleep assessment

Respiratory polygraphy is performed during the first night following stroke onset using a Nox-T3 respiratory polygraph (Nox Medical, Inc., Reykjavik, Iceland), according to standard procedures provided by the manufacturer. This device allows the recording of nasal pressure, snoring, rib cage and abdominal movement, pulse oximetry, activity and body position.

Respiratory polygraphies are analysed by a certified sleep physician immediately on the next day (visit 1, study day 1) to allow inclusion of eligible participants (Fig. 1). Respiratory polygraphies are repeated at visit 6 (day 90 ± 7) to examine the evolution of SDB following a two-week washout period involving a temporary discontinuation of ASV therapy.

### Randomization

Random allocation of patients with significant SDB (*n* ~ 134) (AHI > 20/h) in a 1:1 ratio to the two treatment arms is performed using a list with permuted blocks and stratified for the study centre and baseline AHI (AHI < or ≥ 45/h assessed by respiratory polygraphy in the screening night, visit 0). The trial statistician generated the randomization list. Study physicians randomize patients using the electronic data capturing system after obtaining the informed consent and evaluating all exclusion and inclusion criteria. No study personnel, except the trial statistician, has access to the randomization list until completion of the trial.

### Intervention: adaptive servo-ventilation

The investigational device for ASV in this trial is the “AirCurve™10 CS PACEWAVE” device, (ResMed Ltd., 1 Elizabeth Macarthur Drive, Bella Vista NSW 2153, Australia). It is indicated and approved for clinical use in Switzerland, European Union, and other countries. For this trial, the device is strictly applied in accordance to its approved indications and the instruction manual. Commercially available and approved full-face or nasal masks are used as patient–ventilator–interface to achieve optimal mask fit without relevant leakage. As in clinical routine, all patients will undergo education and training in the use of the device.

In case of an acute upper respiratory tract infection, the intervention will be temporarily or constantly discontinued based on the study physicians and pulmonary physicians’ decision. ASV treatment will be stopped if a patient reports unusual chest pain, severe headache, increased breathlessness or in case of new evidence for congestive heart failure (as defined in the exclusion criteria Table 1) and on patients’ request.

No comparator or sham treatment is administered. SDB patients not randomized to ASV will be treated according to best current medical practice. We decided against sham ventilation due to the following reasons: (1) Sham ventilation may cause even greater distress in patients than effective ventilation and patients will not perceive any benefit in terms of an increase in sleep quality and an improvement in daytime sleepiness. This may lead to a greater decrease in treatment compliance in sham versus effectively treated patients. (2) As described by Tomfohr and colleagues [26], stroke patients’ burden of a ventilation intervention is already high and informing them that they may be randomized to a placebo ventilation will further decrease the number of given informed consents. (3) The use of a subtherapeutic ventilation pressure may act as a partial treatment of nightly occurring apnoeas and hypopnoeas and thus decrease discriminatory power when comparing the two randomized groups.

### Assessment of outcomes

#### Primary outcome: final infarct change from visit 1 to visit 6 on magnetic resonance imaging (MRI)

**Fig. 2:**
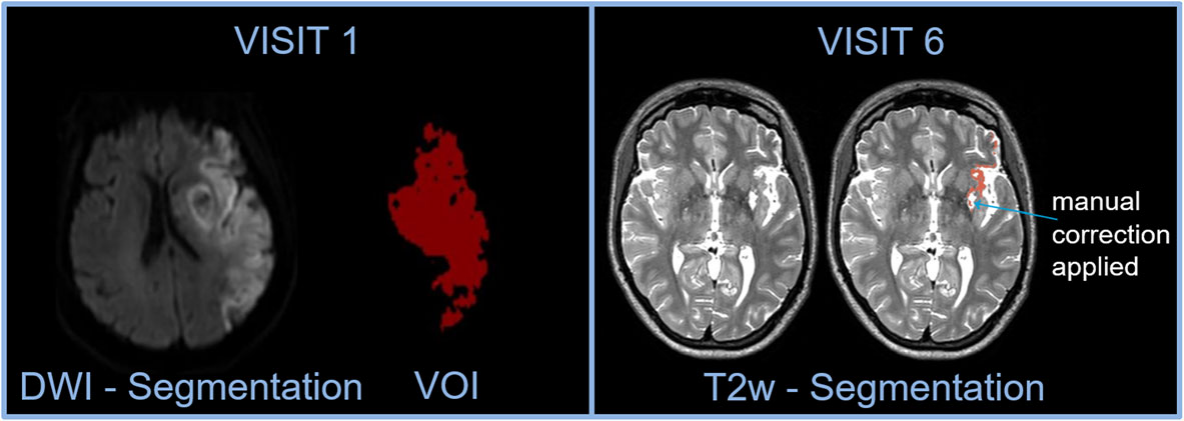
Assessment of primary endpoint. Infarct volume is manually segmented on DWI (diffusion weighted imaging) MRI scans at visit 1, i.e. after potential lysis therapy and before ASV-treatment, and on T2w MRI scans at visit 6. The lesion mask (volume of interest, VOI) is used to preselect the lesion on the T2w images and manual correction is then applied

The infarct volumes (measured in cm^3^) are assessed at visit 1 and visit 6 (see Table 2 displaying which assessments are performed at which visits, according to [27]). We measure infarct volume on DWI (diffusion weighted imaging) MRI scans at visit 1, i.e. after potential lysis therapy and before ASV-treatment, and on T2w MRI scans at visit 6 (see Fig. 2 for illustration). Two independent raters blinded regarding patients’ treatment allocation and presence of SDB assess the primary MRI outcome supervised by two senior neuroradiologists. Change of lesion volume from visit 1 to visit 6 is compared between the treatment groups (SDB ASV+ and SDB ASV−). Change of lesion volume from visit 1 to visit 6 will also be compared between the patient groups with sleep-disordered breathing (SDB ASV+ and ASV− together) and without sleep-disordered breathing (noSDB).

**Table 2:**
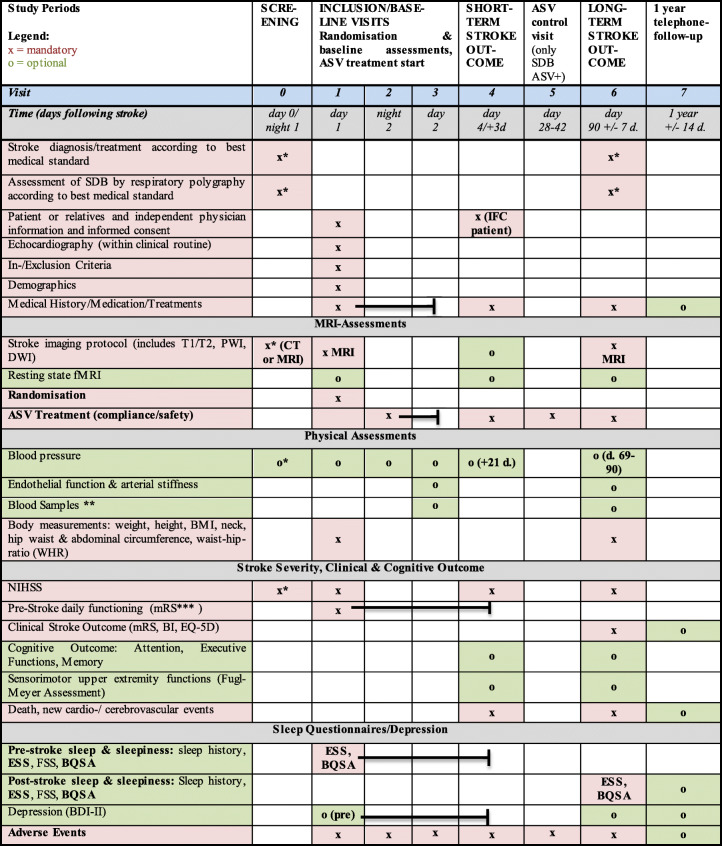
Summary of assessment at each time point according to SPIRIT guidelines

*The following assessments will be collected as long as they are performed within the clinical routine. **Routine analyses: total cholesterol, LDL, HDL, triglycerides, glycated haemoglobin (HbA1c), glucose, C-reactive protein (CRP), thrombocytes, INR, PTT and fibrinogen. Inflammatory markers (optional, depending on funding): MMP-9, TIMP-1, TIMP-4 and sVCAM-1. ***Clinical baseline. Black bars indicate that an assessment can be performed within the indicated timeframe

#### Secondary outcome measurements

##### Demographics

Study physicians and stroke neurologists collect patients’ demographical data during the first 1–2 days following stroke.

##### Stroke data/localization/treatment

At visit 1, study physicians record stroke-specific data such as time of stroke and stroke localization. Antithrombotic treatment before admission (anticoagulation, antiplatelet agents, antihypertensive drugs, cardiac drugs) and thrombolysis at admission is noted. Additional assessments of pre-stroke cardiovascular risk factors are performed: family history, arterial hypertension (BP ≥ 140/90 mmHg measured ≥ 3 times before stroke or patients under treatment for hypertension), diabetes (fasting glucose level ≥ 140 mg/dL or patients treated for diabetes), smoking status, alcohol intake, hypercholesterolemia (cholesterol level ≥ 250 mg/dL or previously under statins) and previous history of coronary heart disease or heart failure, and a validated cerebrovascular risk score (“the Essen Stroke Risk Score, ESRS”) will be determined [28].

##### Medical history including medication

Study physicians record the pre-stroke medical history including medication and treatments at discharge. Assessment of post-stroke medical history including medication is repeated during visit 6 and visit 7 and includes all relevant diseases that (i) have been diagnosed by a physician and/or that (ii) have been treated (pharmacologically or non-pharmacologically).

##### Assessment of stroke severity and clinical outcome

- Stroke severity is assessed by means of the NIHSS at admission (visit 0), after 24 h (visit 1), at patient’s hospital dismissal (visit 4) and at the 90 ± 7 days’ follow-up during visit 6.
- Modified Rankin Scale (mRS) [29] is used to characterize patient’s (in)dependence in daily functioning and in performance of daily activities before the stroke and will be assessed before hospital discharge (between visits 1 and 4).
- Clinical stroke outcomes are assessed at visit 6 and visit 7 using the Barthel Index (BI) [30] and the mRS [29] as well as patient’s subjective rating about quality of life (EQ-5D-3L) [31].
- Sensorimotor upper extremity functions are assessed by the Fugl-Meyer Assessment of upper extremities, which has provided to be reliable and sensitive to assess upper-extremity recovery following stroke at 4 to 7 days after stroke and at 3-month follow-up (visit 4 and 6) [32, 33].
- Death and new cardio-/cerebrovascular events: a composite endpoint of death from any cause, stroke, transient ischaemic attack (TIA), nonfatal myocardial infarction, unplanned hospitalization (or unplanned prolongation of a planned hospitalization) for heart failure or unplanned hospitalization (or unplanned prolongation of a planned hospitalization) leading to urgent revascularization is assessed at visit 4, visit 6, and visit 7 (see Additional file, table 1 for a definition of the events)
- Cognitive outcomes: Cognitive performance measures, assessed by neuropsychological tests (see Additional file, table 2 for details) of language, neglect, attention, executive functions and verbal and visual short-and long-term memory, are obtained at visit 4 and visit 6.

##### Physiological assessments

- Body measurements: Body weight-related measures are assessed at visit 1 and visit 6. Measures include: height in metre and body weight in kg in order to calculate BMI (kg/m^2^), neck circumference, waist (taken at the narrowest waist level) and hip (taken at the level of hip bones) circumference to calculate the Waist/Hip-Ratio (WHR), abdominal circumference (taken at the level of the umbilicus).
- Blood pressure (BP): (1) acute phase: supine BP is measured at the non-paralyzed arm using a non-invasive BP monitoring device at each site’s stroke unit. Mean systolic and diastolic BP values and minimal and maximum values are calculated for the individual patients. BP variability is calculated by standard deviation, coefficient of variation and maximum minus minimum of both diastolic and systolic values. Night and day parameters can be analysed separately. (2) At hospital dismissal (after visit 4) and at 3 months following stroke (before visit 6): a 3-week BP-monitoring is performed with an ambulatory device (Boso Medicus PC 2, Bosch & Sohn, Germany). Measurements are performed up to 3 times successively, 3 times a day (morning, noon, evening) in a sitting position by the patients themselves or the caregivers. The devices themselves store the measurements automatically. At the same time patients are filling in a diary. Mean systolic and diastolic BP values and minimal and maximum values are calculated for the individual patients over the whole period. BP variability is calculated as mentioned above.
- Endothelial functioning and arterial stiffness: endothelial function and arterial stiffness is assessed with peripheral arterial tonometry (PAT) (EndoPAT 2000, ItamarMedical Ltd., Caesarea, Israel) in the acute phase at visit 3 and the chronic phase at visit 6 as described in Patvardhan and colleagues [34]. All calculated parameters are summarized in the Additional file, table 3.
- Blood coagulation parameters and inflammatory blood markers: blood is sampled from a venepuncture for routine and complementary blood tests at visit 2 and 6. Blood sampling for routine analysis of total cholesterol, low- and high-density lipoprotein (LDL and HDL) triglycerides, glycated haemoglobin (HbA1c), glucose, C-reactive protein (CRP), thrombocytes, international normalized ratio (INR), partial thromboplastin time (PTT) and fibrinogen is done according to the hospital protocol and analysed locally. Blood samples of approximately 50 mL are obtained from each patient. Optionally (depending on funding), remaining blood samples will be stored at each site for up to 15 years to be reanalysed for inflammatory markers: MMP-9, TIMP-1, TIMP-4 and sVCAM-1. In the informed consent form, patients are informed and agree with the storage of biological specimens, if applicable.

##### Assessment of ASV treatment compliance

Compliance to ASV treatment is checked daily during the initiation of ASV treatment using the devices internal recordings. Thereafter, verification of compliance takes place at clinical controls at visits 2, 4, 5 and 6. Sufficient compliance is defined as a cumulative ASV use in ≥ 70% of all nights and ≥ 4 h per night in the first 3 months after stroke. All patients who started ASV treatment/training will be analysed as ASV patients (intention to treat; ITT). For secondary analyses, patients with SDB are stratified according to their compliance: compliant patients (per protocol, PP) will be compared to non-compliant participants and to participants who did not receive ASV-treatment.

##### Pre- and post-stroke sleep history and sleep questionnaires

- Pre-stroke sleep history: at days 1 to 4 following stroke, patients are shortly interviewed about pre-stroke sleep problems. Interview questions include (1) questions about usual bedtime, sleep latency, rise time and sleep duration during the week and on the weekends within the last month before stroke, (2) bed- and rise time and sleep duration during the two nights before stroke, (3) daytime naps the 2 days before stroke and (4) any previous significant sleep disturbances within the last month before stroke and intake of medication.
- Post-stroke sleep history is assessed at visit 6 and 7 and include (1) questions about usual bedtime, sleep latency, rise time and sleep duration during the week and at the weekends within the last month from now and (2) occurrence of any significant sleep disturbances within the last month and intake of medication.
- Sleep and depression questionnaires: all stroke patients fill in the following questionnaires to measure sleepiness/fatigue, likelihood for sleep apnoea and symptoms of depression at visits 1, 6 and 7:
  1. Epworth Sleepiness Scale (ESS) [35].
  2. Fatigue Severity Scale (FSS) [36]
  3. Berlin Questionnaire for Sleep Apnoea (BQSA) [37]
  4. Beck Depression Inventory (BDI-II) (validated German translation of [38])

Data collection of primary and secondary outcome measurements continues even if a patient of the ASV+ group discontinues ASV treatment. The only condition when data collection stops is if patients withdraw their informed consent. Data collected until this point in time will be kept and analysed as agreed by patients signing the informed consent forms.

### Data management and study monitoring

The case report forms (CRFs) in this trial are implemented electronically using a dedicated electronic data capturing (EDC) system (REDCap). The EDC system was activated for the trial only after successfully passing a formal test procedure. All data entered in the electronic CRFs are stored on a Linux server in a dedicated mySQL database.

Study monitoring including source data verification of a selected subject at each centre is performed by the Clinical Trial Unit (CTU) of the University of Bern, Switzerland. With the informed consent, patients agree that relevant data can be viewed and shared with the research team that initiated the study (coordinating research team in Bern), with the ethical and regulatory authorities (including the representative of the trial’s insurance in case of a potential study-related damage) and the trial’s monitors.

### Statistical analysis

Descriptive summary statistics for efficacy and safety variables will be presented for the entire population and by study arm (no SDB patients, and SDB patients randomized to the ASV+ and ASV- arms). Inferential statistical methods will be used to highlight interesting aspects of the data and primarily for the comparison of outcome variables in the two randomized arms. In addition, comparisons of selected outcomes will be carried out between the two cohorts. The significance level of all statistical tests will be 5% and no control for multiplicity will be applied. Corresponding 95% confidence intervals will be presented as appropriate.

Two main analysis populations will be used:

- *Full Analysis Set (FAS)*: this population will include all enrolled patients. In line with the intent-to-treat principle, randomized patients will be analysed according to the treatment group assigned at randomization. The FAS will be the main analysis population for efficacy and safety variables. In particular, it will be the primary population for the comparative analysis of the two randomized treatment groups.
- *Per-protocol (PP) population*: it will include randomized patients fulfilling all selection criteria and sufficiently compliant with the protocol. The list of criteria, leading to exclusion from the PP analysis, which, among other criteria, will consider the compliance with ASV procedures, will be finalized before database closure for the primary analysis.

The comparative analysis of infarct volume data will be performed using an analysis of covariance model with adjustments at least for the stratification variables used at randomization. In case of violation of the statistical validity conditions for the model, a suitable non-parametric alternative model will be employed, such as the Van Elteren test.

Between-arm comparisons of continuous variables will be performed using the same methodology as for the primary variable, whereas models may include additional explanatory variables, such as the baseline value of specific outcome variables assessed at study end. Logistic regression will be used for comparisons based on binary variables.

Furthermore, similar models will be used to compare outcome variables between patients from the two cohorts of randomized patients and no SDB patients. Due to the non-randomized design and the definition of the cohorts, the set of explanatory variables included in these models will be different: AHI will not be included, but other potential confounders, such as age and gender may be. No interim analysis of efficacy endpoints is planned. Primary and secondary efficacy outcomes will be analysed after all randomized patients have completed the study or have left it prematurely. Interim analyses of safety may be performed. The analysis of safety data will use descriptive statistical methods. Deviations from the analyses outlined in this section and in the statistical analysis plan will be listed and justified in the Clinical Study Report. No imputation of missing variables will be employed in the primary comparative analysis of efficacy parameters in the two randomization groups. For supportive analyses, imputation strategies considered conservative may be used.

### Power calculation and sample size

Based on feasibility considerations, approximately 134 patients are expected to be randomized in a 1:1 proportion into the group with ASV treatment and without ASV treatment (SDB ASV+ and SDB ASV-). Based on a two-sided *t* test at the 5% significance level, this sample size will provide at least 80% power to detect an effect size (Cohen’s d) of 0.49 for the between-group difference in primary outcome (difference in infarct size from visit 1 (day 1) to visit 6 (day 90)). Based on preliminary investigations in a similar patient pool, the standard deviation is assumed to be 20 cm^3^ (unpublished data), so that the detectable effect size corresponds to a between-arm difference of approximately 10 cm^3^. In secondary analyses, the outcomes will be compared in patients with and without SDB: the detectable difference is expected to be approximately 10 cm^3^ also for this analysis. Calculations were performed using the statistical software package R (https://www.r-project.org/) [39]. These differences are considered meaningful from a clinical point of view.

Based on these considerations, the planned sample size is considered adequate to address the main study objective.

### Safety

Serious adverse events (SAE), serious adverse device effects (SADE) and serious adverse incidents (SAI) are assessed at each centre and reported to the centres’ local ethical committees according to their requirements and within 24 h of awareness to the coordinating centre in Bern.

The coordinating centre in Bern also submits an annual safety report to their ethical committees and to centres’ principal investigators (PI). All PIs are responsible to continuously evaluate the risk-benefit profile of the intervention for each examined patient. Findings or available data indicating an imbalanced risk-benefit profile must be reported to the ethical authorities and the PIs must take the necessary steps.

In case of a SAI, each centre will contact the responsible person for medical device vigilance in order to decide whether reporting to the national health surveillance authorities according to the legal requirements at each centre is indicated.

## Discussion

eSATIS trial is the first trial to assess the impact of immediate treatment of significant SDB (AHI > 20/h) in acute ischaemic stroke patients with non-invasive ASV on infarct progression in a prospective interventional randomized controlled design. The trial will give information on the feasibility and efficacy of ASV treatment in patients with stroke and SDB and allow assessing the impact of SDB on stroke outcome. Diagnosing and treating SDB during the acute phase of stroke is not yet current medical practice. Evidence in favour of ASV treatment from a randomized multicentre trial may lead to a change in stroke care and to improved outcomes.

Limitations of this trial include the following: (1) The compliance to ASV is expected to be around 50%. This will have an impact on the interpretation of results. (2) The treatment is open label, which may result in a bias affecting the assessment of secondary clinical outcomes. However, sham ventilation may cause even greater distress in patients than effective ASV treatment without beneficial effects on sleep quality and improvement of daytime sleepiness and is ethically questionable. (3) It is unknown whether the sample size of 134 randomized patients will be sufficient to detect the real treatment effect, since no data on the impact of ASV treatment on the evolution of the lesion volume are available so far.

## Trial status

Recruitment started in August 2015 at the coordinating centre of the Bern University Hospital and was successively expanded to the other centres: Cantonal Hospital St. Gallen (Switzerland), Almazov National Medical Research Centre St. Petersburg (Russia), Grenoble Alpes University Hospital (France), Medical Centre of the Johannes Gutenberg University, Mainz (Germany) and Charité - Universitätsmedizin Berlin (Germany). Recruitment is expected to be completed in July 2021. The current version of the protocol is version 4.1 dated 07.02.2017.

## Supplementary Information

**Additional file 1.**

## Data Availability

Not applicable as the study is ongoing. After study completion and publication of the results, the datasets generated and/or analysed during the current study will be available from the corresponding author on reasonable request.

## Abbreviations

AHI: Apnoea-hypopnoea index
ASV: Adaptive servo-ventilation
BDI-II: Beck Depression Inventory II
BI: Barthel Index
BP: Blood Pressure
BQSA: Berlin Questionnaire for Sleep Apnoea
CHF: Congestive Heart Failure
CPAP: Continuous positive airway pressure
CRFs: Case report forms
CSA: Central sleep apnoea
CTU: Clinical Trial Unit
DWI: Diffusion weighted imaging
ECASS: European Cooperative Acute Stroke Study
EDC: Electronic data capturing
ESS: Epworth Sleepiness Scale
FAS: Full analysis set
FSS: Fatigue Severity Scale
ITT: Intention to treat
MRI: Magnetic resonance imaging
mRS: modified Rankin Scale
NIHSS: National Institute of Health Stroke Scale
NYHA: New York Heart Association
OSA: Obstructive sleep apnoea
PI: Principal investigator
PP: Per protocol
SADE: Serious adverse device effects
SAE: Serious adverse events
SAI: Serious adverse incidents
SDB: Sleep-disordered breathing
TIA: Transient ischaemic attack
VOI: Volume of interest
