# Supplementary material for: Effect of early sleep apnoea treatment with adaptive servo-ventilation in acute stroke patients on cerebral lesion evolution and neurological outcomes: study protocol for a multicentre, randomized controlled, rater-blinded, clinical trial (eSATIS: early Sleep Apnoea Treatment in Stroke)
Source: Trials. 2021 Jan 22;22:83. doi: 10.1186/s13063-020-04977-w (PMC7820538; doi:10.1186/s13063-020-04977-w)
Supplement: Supplementary file 1 — Additional file 1. [file 13063_2020_4977_MOESM1_ESM.docx]

**Effect of early sleep apnoea treatment with adaptive servo-ventilation in acute stroke patients on cerebral lesion evolution and neurological outcomes: study protocol for a multicentre, randomized controlled, rater-blinded, clinical trial (eSATIS: early Sleep Apnoea Treatment in Stroke)**

**-**

**Duss SB. and colleagues**

**-**

**ADDITIONAL FILE**

**Table 1. Definitions of new cardio-/cerebrovascular events in composite endpoint**

| Stroke | Rapidly developing clinical symptoms and/or signs of focal, and at times global (applied to patients in deep coma and subarachnoid haemorrhage), loss of cerebral function, with symptoms lasting more than 24 hours or leading to death, with no apparent cause other than that of vascular origin (Definition provided by the World Health Organization (WHO)).  Ischemic and haemorrhagic strokes will be assessed here. |
| --- | --- |
| TIA | Acute loss of focal cerebral or ocular function with symptoms lasting less than 24 hours and which after adequate investigation is presumed to be embolic or thrombotic vascular disease (1, 2). |
| Myocardial infarction | hsTroponin > 99th percentile upper reference limit with at least one of the following: symptoms of ischemia; new or presumed new ST-segment-T wave changes or new left bundle branch block; development of pathological Q waves in the ECG; imaging evidence of new loss of viable myocardium or new regional wall motion abnormality; identification of intracoronary thrombus by angioplasty or autopsy (3). |
| Heart failure | Symptoms and signs typical of heart failure and either reduced left ventricular ejection fraction (<50%) or relevant structural heart disease and/or diastolic dysfunction (4). |
| Urgent cardiac revascularization: | Revascularization will be considered to be urgent when a patient will be admitted to the hospital with persistent or increasing symptoms and the revascularization procedure is performed during the same hospitalization period (5). |

Abbreviations: ECG: electrocardiogram; hsTroponin: high sensitivity Troponin; TIA: Transient ischemic attack; WHO: World Health Organization.

**Table 2. Assessment of cognitive outcomes**

| Domain | Test | Description | Normative values | References |
| --- | --- | --- | --- | --- |
| Language – Aphasia screening | Bern Word Finding test (B-WFT) | This test was developed in the Department of Neurology, Bern University Hospital (Inselspital) and University of Bern, Switzerland. Participants are asked to name 20 black and white line drawings representing living and non-living objects. If they are unable to provide an answer within 10 s, the investigator displays the next drawing. Assessment time is about 5 minutes.  Two parallel versions exist, one of them will be used for the first baseline assessment at V4 and the other version at V6. | Normative data (z-values) are derived from a sample of 52 participants aged between 22 – 81 years. | (6) |
| Neglect | Bells Test | In this cancellation test, the patient is asked to circle with a pencil all target stimuli (35 bells) embedded within distractors (280 houses, horses, etc.). The examiner measures completion time and notes the position of the target stimuli (bells) crossed out by the participant on the diagrammatic plan. Test and time measurement are stopped when the patient claims to have found all target stimuli.  This test will be performed at V4 and will be repeated at V6. No alternate version will be used, since no learning effects are expected. | 4 and more omissions = evidence for attention deficit  (Healthy controls do 0-3 omissions)  6 and more omissions in the left three columns = evidence for a neglect | (7, 8) |
| Attention | Test of Attentional Performance | *Alertness*: refers to general wakefulness and enables a person to respond quickly and appropriately to any given demand. It is the basis of every attention performance. In this test, reaction time is examined under two conditions (execution time 4.5 min.):   1. Simple reaction time measurements: a cross appears on the monitor at randomly varying intervals and the participant should respond as quickly as possible by pressing a key. Intrinsic alertness is measured in this condition. 2. In a second condition, a tone precedes the white cross on the screen and participants are asked to press a button to the appearance of the cross only. ("phasic arousal").   *Go/No-Go Task:* assessed the capability to inhibit an inadequate response. The task is to press a key as fast as possible when the white cross appears on the screen but to withhold response if a plus sign appears (condition 1, execution time 2 min.).  These tests are performed at V4 and repeated at V6. | For the tests of alertness and inhibition (go/no-go task), reference values from 19 to 90 years of age are provided by the authors of the TAP. | TAP 2.3: <http://www.psytest.net/>  (9) |
|  | Psychomotor Vigilance Test (PVT) | *Sustained or vigilant attention:* the task is to press a button to a light stimulus that appears at an irregular interstimulus interval. The reaction time of the participant is then displayed on the screen (execution time 10 min.). Reaction times and lapses mainly will be compared within patients from the baseline and outcome assessment and between the three patient groups.  This test will are at V4 and will be repeated at V6. |  | (10-13)  PVT (http://bhsai.org/downloads/pc-pvt/) |
| Executive functions | Trail Making Test (version A and B) | This test measures attention, working speed and mental flexibility and is divided into two parts.   1. Part A: digits ranging from 1 to 25 are printed on a sheet of paper in a randomly distributed order. Participants are asked to connect these digits as fast as possible with a straight line in ascending order. 2. Part B: digits (1-13) and letters (A-L) are printed on a sheet of paper in a randomly distributed order. Participants are asked to alternately connect numbers and digits in ascending order as fast and accurate as possible. | Age-stratified normative data are provided by Tombaugh and colleagues (14) derived from a large Canadian sample of 858 individuals aged 20-89 years and by Rodewald and colleagues (15) derived from a large German speaking sample of 405 individuals aged 18-84 years. | (14-16), |
|  | Victoria Stroop | This test examines participants’ inhibition and interference control.   1. Run 1 (colors): participants are asked to name the color of colored dots. 2. Run 2 (word): they are asked to name the print color of prepositions words (e.g. yellow if “and” is printed in yellow). 3. Run 3 (interference): includes color-words (such as red, blue, green, yellow), however they are printed in a color which is incongruent to their name or meaning (for example, the yellow word is printed with green color). Participants should rapidly name the color of the words and suppress their impulse to read the color-word.   Naming time is measured. | Age-group stratified norms (18-94 years divided into 9 groups) will be taken from Troyer, Leach & Strauss (17). | (18) |
| Short-term verbal and visual memory | Digit Span Task forward | Participants listen to a series of digits (e.g., '8, 3, 4') and are asked to immediately repeat them back. Following at least one out of two successful repetition, they are given a longer list (e.g., '9, 2, 4, 7'). The participant’s digit span represents the longest series of digits he or she can correctly repeat. | Age- and education-stratified normative values are provided by Balzer and colleagues (19). | (19) |
|  | Digit Span Task backward | Participants listen to a series of digits (e.g., '4, 2’') and are asked to immediately repeat them in backward order (correct answer: e.g., ‘2, 4’). Following at least one out of two successful repetition, they are given a longer list (e.g., '8, 3, 6') (correct answer: ‘6, 3, 8’). The participant’s digit span represents the longest series of digits he or she can correctly repeat. | Age-stratified normative values are provided by Wechsler Memory Scale – Revised | (20) |
|  | Corsi-block Task | The Corsi block-tapping task assesses [visuo-spatial](http://en.wikipedia.org/wiki/Visual-spatial_ability) [short-term memory](http://en.wikipedia.org/wiki/Short_term_memory). The investigator taps a sequence of up to nine identical spatially separated blocks. Participants are asked to mimic this sequence. The sequence starts out simple, with only three blocks, but becomes more complex until the participant fails to reproduce two sequences in a row. | Age- and education-stratified normative values are provided by Balzer and colleagues (19). | (19) |
| Long-term verbal and visual memory | Hopkins Verbal Learning Test – Revised | This test assesses verbal long-term memory for words. Within one learning trial, a list of 12 words are read to the participants. Their task is to recall these words after every learning trial. Following 3 learning trials and after a delay of 25 minutes participants are again asked to recall the words (free recall).  Afterwards a recognition test is performed. The same 12 words are intermixed with new distractor words and participants have to indicate for each word whether it is old or new. Administration duration is between 5-10 minutes with a delay of 20 minutes. 6 different test forms are available. To prevent retest-effects due to long-term retrieval, two different test forms are administrated at V4 and at V6. | Normative data is available for participants between the age of 16-92. | (21) |
|  | Brief Visuospatial Memory Test – Revised | This test assesses visuospatial memory in participants. Within 3 learning runs, participants are shown a card with 6 geometric shapes for 10 s and are asked to remember them and to draw them by heart following each run. After a 25 min. break, they are again asked to remember and draw the previously memorized geometrical shapes. Total administration time is about 15 minutes. To prevent retest-effects due to long-term retrieval, two different test forms are administrated at V4 and at V6. | Normative data is available for participants between the age of 18-79 years. | (22) |

Notes: V4 stands for visit 4 (4 – 7 days following stroke) and V6 for visit 6 (90+/-7 days following stroke).

To consider for administration:

- In case of an aphasia affecting speech production but not speech comprehension only the following non-verbal tests are performed: Alertness, Go/No-Go task, Psychomotor Vigilance Test (PVT), trail making test, visual short- and long-term memory tests (Corsi block-tapping test, Brief Visuospatial Memory Test – revised).
- In case of an aphasia affecting speech comprehension no neuropsychological test are performed. In patients with a neglect, all three attention tasks and the verbal short- and long-term memory tasks are performed.

**Table 3. Calculated parameters using peripheral arterial tonometry (PAT) (EndoPAT 2000, ItamarMedical Ltd, Caesarea, Israel)**

| **Reactive Hyperemia Index (RHI) and LnRHI (natural log of RHI)** | A measure of endothelial function. It is calculated by the post-to-pre occlusion PAT signal ratio in the occluded arm, relative to the same ratio in the control arm. It is corrected for baseline vascular tone of the occluded arm (Normal RHI >1.67 LnRHI > 0.51; Abnormal RHI ≤ 1.67 or LnRHI ≤ 0.51). LnRHI provides a better double-sided distribution of physiological values close to a Gaussian distribution. |
| --- | --- |
| **FRHI (Framingham Heart Study Index)** | Index first calculated in the Framingham Heart Studies (see e.g., (23) ). It is a natural log transform of the post- to pre-occluded PAT amplitudes relative to the same ratio of the PAT amplitudes measured at the control arm. It does not include a baseline correction and uses a shorter post occlusion time (1.5 - 2 Minutes). |
| **Augmentation Index (AI)** | A measure of arterial stiffness, calculated based on a pulse wave analysis of the signal measured by EndoPAT. Arterial stiffness is an independent risk factor for cardio-cerebrovascular events, and is not necessarily correlated with endothelial function. AI is calculated from the PAT pulse by finding the systolic peak P1 and the backward reflected peak P2 as (P2-P1)/P1. The AI_@75_ is heart rate corrected to a standard HR of 75 BPM. |
| **Heart rate variability (HRV)** | Calculated from the PAT signals during baseline recording (pre-occlusion period) that must have a minimal duration of 5.5 minutes (we recorded 6 minutes in the eSATIS Study). |

**ADDITIONAL REFERENCES**

1. Hatano S. Experience from a multicentre stroke register: a preliminary report. Bulletin of the World Health Organization. 1976;54(5):541-53.

2. Special report from the National Institute of Neurological Disorders and Stroke. Classification of cerebrovascular diseases III. Stroke. 1990;21(4):637-76.

3. Thygesen K, Alpert JS, Jaffe AS, Simoons ML, Chaitman BR, White HD. Third universal definition of myocardial infarction. Nature reviews Cardiology. 2012;9(11):620-33.

4. McMurray JJ, Adamopoulos S, Anker SD, Auricchio A, Bohm M, Dickstein K, et al. ESC guidelines for the diagnosis and treatment of acute and chronic heart failure 2012: The Task Force for the Diagnosis and Treatment of Acute and Chronic Heart Failure 2012 of the European Society of Cardiology. Developed in collaboration with the Heart Failure Association (HFA) of the ESC. European journal of heart failure. 2012;14(8):803-69.

5. De Bruyne B, Fearon WF, Pijls NH, Barbato E, Tonino P, Piroth Z, et al. Fractional flow reserve-guided PCI for stable coronary artery disease. The New England journal of medicine. 2014;371(13):1208-17.

6. Berger E-M. Normierung eines Benenn- und semantischen Entscheidungstests für biologische und manipulierbare Objekte in deutscher Sprache. : Unpublished Masterthesis. University of Bern.; 2012.

7. Gauthier L, Dehaut F, Joanette Y. The Bells Test: A quantitative and Qualitative Test for Visual Neglect. International Journal of Clinical Neuropsychology. 1989;2:49-54.

8. Ferber S, Karnath HO. How to assess spatial neglect - line bisection or cancellation tasks? Journal of Clinical and Experimental Neuropsychology. 2001;23(5):599-607.

9. Zimmermann P, Fimm B. TAP. Testbatterie zur Aufmerksamkeitsprüfung.: Vera Fimm, Psychologische Testsysteme; 2012.

10. Dinges DF, Powell JW. Microcomputer analyses on performance on a portable, simple visual RT task during sustained operations. Behavior Research Methods, Instruments, & Computers. 1985;17(6):652-5.

11. Basner M, Dinges DF. Maximizing sensitivity of the psychomotor vigilance test (PVT) to sleep loss. Sleep. 2011;34(5):581-91.

12. Khitrov MY, Laxminarayan S, Thorsley D, Ramakrishnan S, Rajaraman S, Wesensten NJ, et al. PC-PVT: a platform for psychomotor vigilance task testing, analysis, and prediction. Behavior research methods. 2014;46(1):140-7.

13. Reifman J, Kumar K, Khitrov MY, Liu J, Ramakrishnan S. PC-PVT 2.0: An updated platform for psychomotor vigilance task testing, analysis, prediction, and visualization. Journal of neuroscience methods. 2018;304:39-45.

14. Tombaugh TN. Trail Making Test A and B: normative data stratified by age and education. Archives of clinical neuropsychology : the official journal of the National Academy of Neuropsychologists. 2004;19(2):203-14.

15. Rodewald K, Bartolovic M, Debelak R, Aschenbrenner S, Weisbrod M, Roesch-Ely D. Eine Normierungsstudie eines modifizierten Trial Making Tests im deutschsprachigen Raum. Zeitschrift für Neuropsychologie. 2012;23(1):37-48.

16. Strauss E, Sherman EMS, Spreen O. A compendium of neuropsychological tests. Administration, norms, and commentary. Oxford: University Press; 2006.

17. Troyer AK, Leach L, Strauss E. Aging and response inhibition: Normative data for the Victoria Stroop Test. Neuropsychology, development, and cognition Section B, Aging, neuropsychology and cognition. 2006;13(1):20-35.

18. Regard M. Cognitive rigidity and flexibility: A neuropsychological study. . University of Victoria: Unpublished Ph.D. Dissertation 1981.

19. Balzer C, Berger J-M, Caprez G, Gonser A, Gutbrod K, Keller M. Materialien und Normwerte für die neuropsychologische Diagnostik. Rheinfelden: Verlag Normdaten; 2011.

20. Wechsler D. Wechsler Memory Scale – Revised Edition. Manual. New York: The Psychological Corporation; 1987.

21. Brandt J, Benedict RHB. Hopkins Verbal Learning Test - Revised. Professional Manual. Lutz, Florida: PAR; 2001.

22. Benedict RHB. Brief Visuospatial Memory Test - Revised. Professional Manual. Florida USA: PAR; 1997.

23. Rubinshtein R, Kuvin JT, Soffler M, Lennon RJ, Lavi S, Nelson RE, et al. Assessment of endothelial function by non-invasive peripheral arterial tonometry predicts late cardiovascular adverse events. European heart journal. 2010;31(9):1142-8.
